# A Rare Case of Parapharyngeal Abscess in a Healthy Adult: Importance of Timely Diagnosis and Intervention

**DOI:** 10.7759/cureus.86158

**Published:** 2025-06-16

**Authors:** Isabel M Gossen, Zachary McSween, Safaa Kamel, Abhinaya R Garlapati, Roxana Lazarescu

**Affiliations:** 1 Internal Medicine, Wyckoff Heights Medical Center, New York, USA; 2 Internal Medicine, St. George's University School of Medicine, True Blue, GRD

**Keywords:** deep neck infection, group a strep sepsis, i&d- incision and drainage, parapharyngeal abscess, upper airway compromise

## Abstract

Parapharyngeal abscesses are rare infections of the neck fascia, most common in young male children. These bacterial infections are difficult to treat due to the complex vasculature in the parapharyngeal spaces. Common complications of untreated abscesses include airway obstruction, internal jugular vein thrombosis, and septic shock. A 43-year-old male presented to the emergency department with a new onset of fever, dyspnea, and sore throat with odynophagia. A bacterial infection was suspected after a physical exam indicated swelling, erythema, and tenderness over the submandibular/parotid regions. Laboratory findings also indicated moderate leukocytosis of 18,000/µL. A subsequent clinical diagnosis of a parapharyngeal abscess was made post-contrast-enhanced computed tomography (CT) scan of the neck. Treatment was initiated with clindamycin, intravenous dexamethasone, and Toradol for analgesic relief. Otolaryngology consultation suggested a transoral incision and drainage (I/D) under general anesthesia. I/D was successful with 3 mL of Group A Streptococcus-laden purulent fluid aspirated. Post-procedure recovery was complication free with significant improvement in laboratory findings and symptoms. The patient’s infection was resolved, and thus, further imaging was not required. A prophylactic seven-day course of oral Augmentin (amoxicillin-clavulanate) was prescribed at discharge. This case provides an excellent investigation into the importance of early intervention for rare bacterial infections of the head/neck region occurring in a patient population with no relevant risk factors.

## Introduction

Parapharyngeal abscesses are rare but potentially life-threatening deep-neck infections, particularly when they cause airway obstruction [1]. Although more common in children under five, especially males [2], they remain challenging due to their deep location and the complex anatomy of the parapharyngeal space, which houses critical structures like the carotid artery, internal jugular vein, cranial nerves, and muscles [3,4]. The parapharyngeal space is a deep neck space, bounded by the skull base superiorly and the hyoid bone inferiorly. It is divided into prestyloid and poststyloid compartments by the styloid process [4]. This anatomical complexity facilitates rapid infection spread, increasing the risk of airway compromise [2,3]. Early recognition and intervention are crucial for managing these infections, but diagnosis and treatment can be difficult.

The etiology of parapharyngeal abscesses is often polymicrobial, involving pathogens such as streptococci, staphylococci, *Haemophilus influenzae*, and oral anaerobes [5]. These infections typically arise from oropharyngeal conditions like dental infections, acute tonsillitis, or peritonsillar abscesses, which spread through the superior constrictor muscle into the parapharyngeal space [5]. Common symptoms include respiratory distress, dyspnea, stridor, trismus, and limited cervical neck extension [1,2]. Diagnosis generally involves clinical evaluation and imaging, with CT or MRI being essential for assessing the severity of deep neck infections, even in the absence of typical symptoms like fever or respiratory distress [1]. Preoperative CT findings, including the infection's distribution and its relationship to the airway, are critical for risk assessment and airway management decisions [1].

Clinicians can often manage cases conservatively with antibiotics, but they may need to perform surgical drainage and intubation when airway involvement is significant or when conservative treatment fails [1]. A combination of intravenous antibiotics and surgical drainage is typically required for management [2]. In this report, we present a case of a 43-year-old male with a left parapharyngeal abscess complicated by epiglottitis and significant airway narrowing. This case emphasizes the importance of timely intervention, multi-disciplinary management, and close monitoring in cases with atypical symptoms, offering valuable insights into managing deep neck infections with potential airway compromise.

## Case presentation

This case involves a 43-year-old man presenting to the emergency department with a two-day history of sore throat, fever, pain with swallowing, and mild dyspnea when lying down. The patient denied vomiting, drooling, recent dental procedures, trauma, or travel history. On examination, the patient appeared febrile and in moderate distress. Vital signs revealed a temperature of 101.8°F, heart rate of 110 bpm, blood pressure of 132/84 mmHg, and oxygen saturation of 96% on room air. Physical examination showed severe swelling and tenderness over the left parotid and submandibular regions, accompanied by erythema and warmth. Additionally, medial displacement of the left tonsil and uvula was noted. There were no visible signs of respiratory distress. Laboratory studies revealed leukocytosis with a left shift, with elevated C-reactive protein (CRP) and elevated blood glucose level. The patient was admitted to the hospital, and initial management included intravenous dexamethasone, clindamycin, and pain control with toreador. Sepsis protocol was activated due to leukocytosis, elevated heart rate, and suspected source of infection.

Considering the patient’s clinical presentation of fever, sore throat, neck swelling, and odynophagia, initial differential diagnoses include peritonsillar abscess, epiglottitis, suppurative parotitis, and acute bacterial pharyngitis. Due to the risk of airway involvement, prompt imaging was warranted.

**Figure 1:**
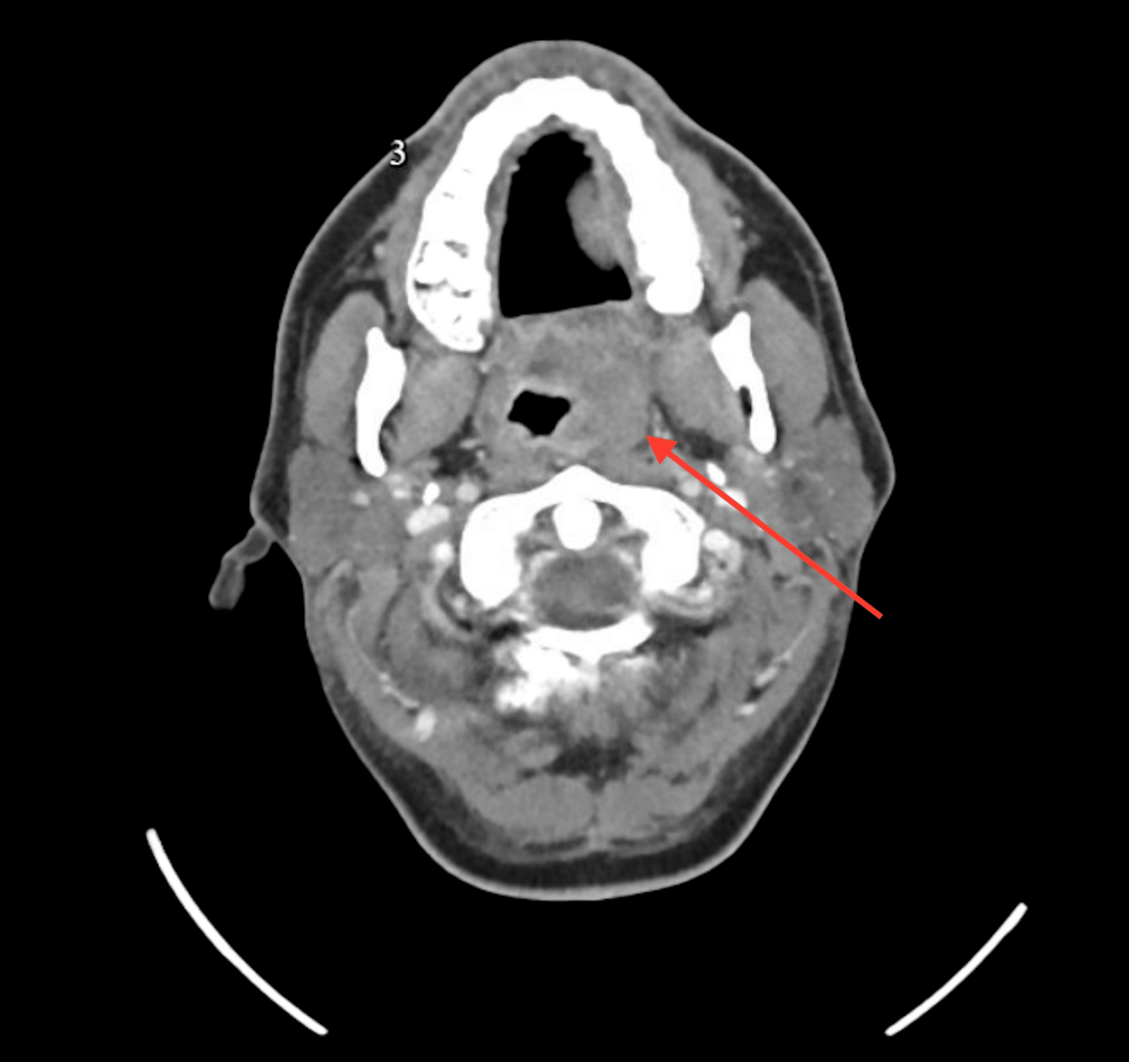
Contrast-enhanced axial CT of the neck demonstrating hypertrophy of lymphoid tissue and a parapharyngeal abscess on the left measuring 2.0 x 1.8 cm indicated by the arrow.

**Figure 2:**
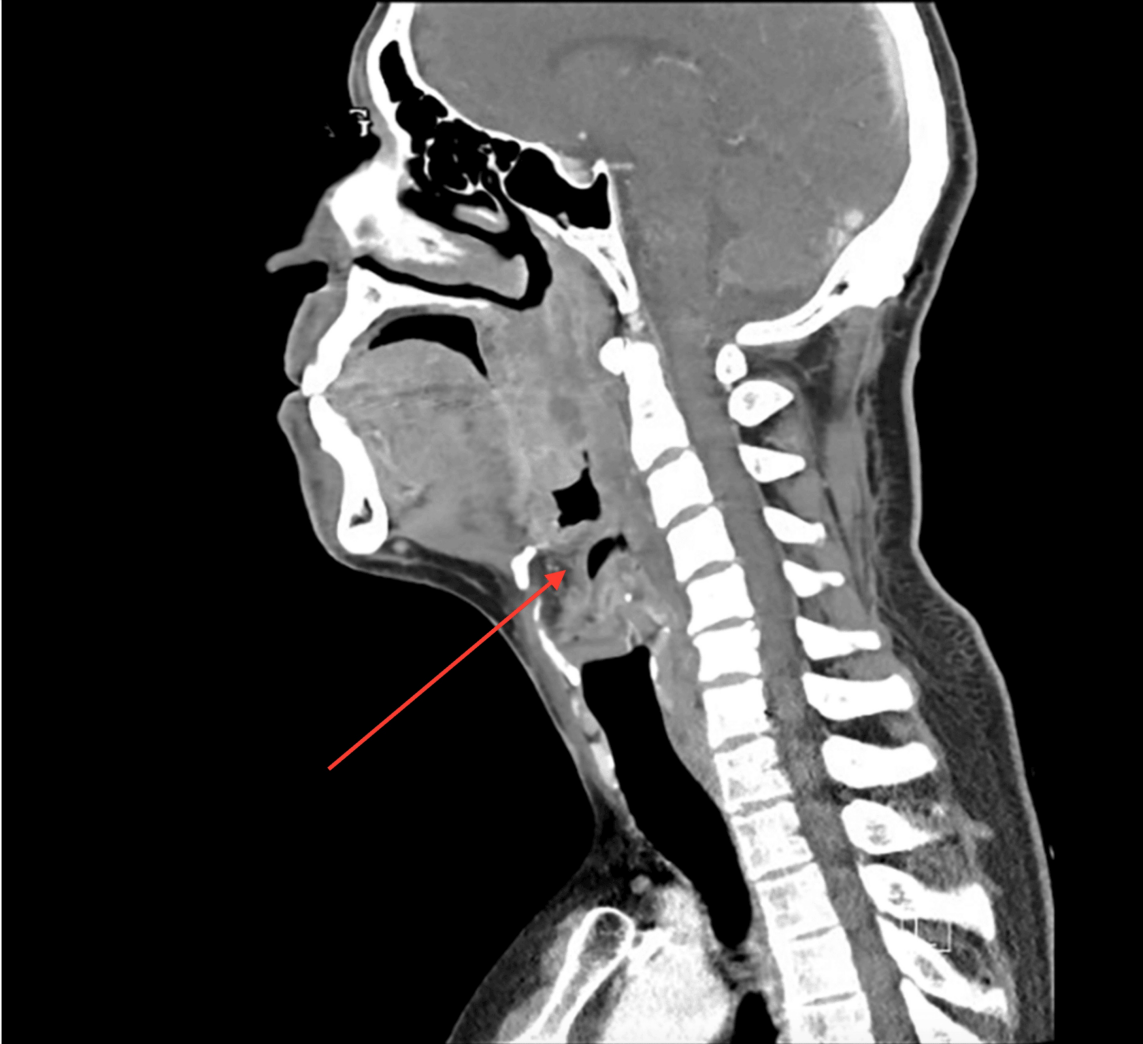
Contrast-enhanced sagittal CT of the neck demonstrating thickening of the epiglottis indicated by arrow.

**Figure 3:**
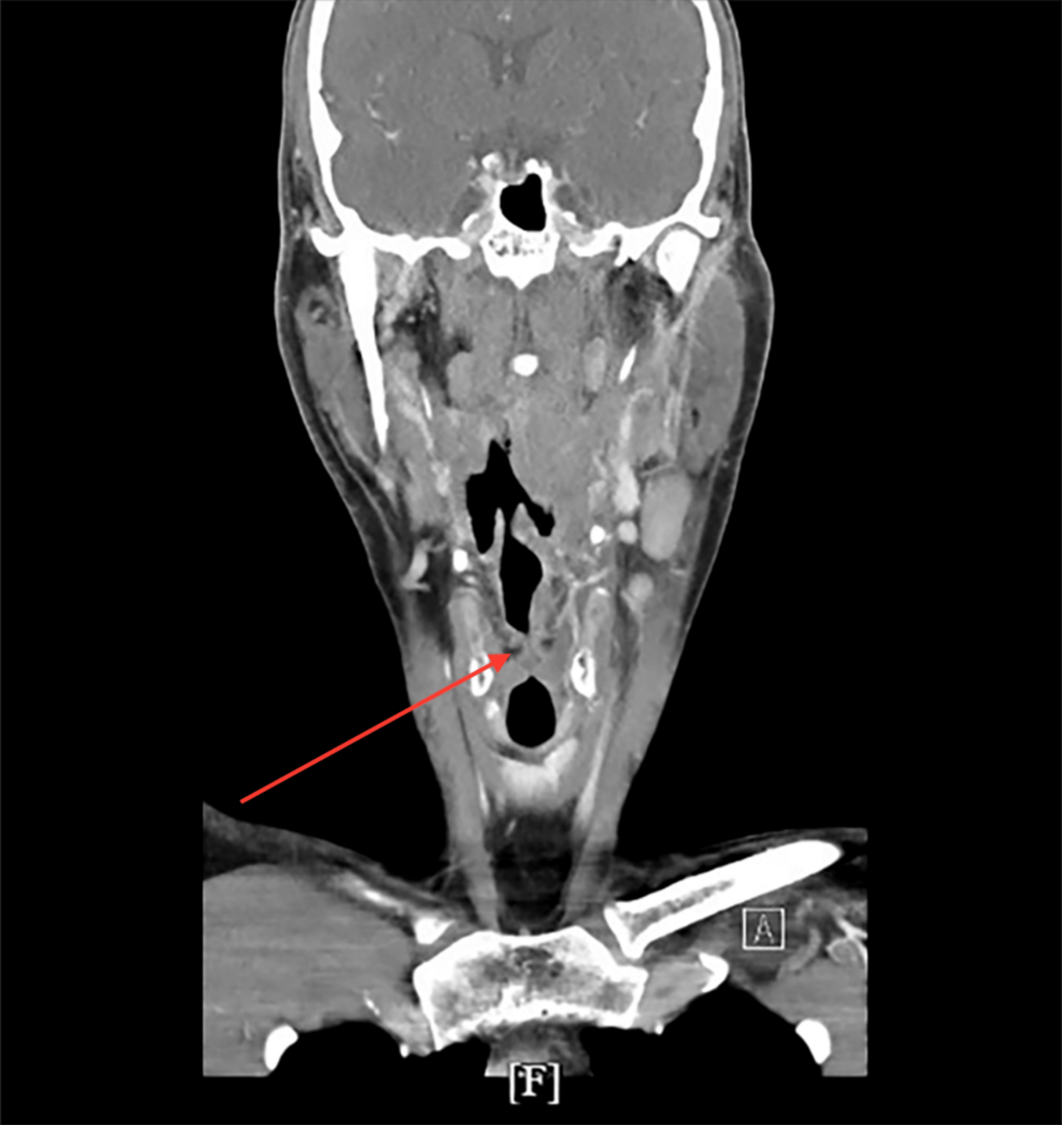
Contrast-enhanced coronal CT of the neck demonstrating thickening vocal cords and walls of the larynx with narrowing of the airway indicated by arrow.

CT of the neck identified a 2.0 x 1.8 cm abscess in the left parapharyngeal space, severe pharyngeal edema, left tonsillar swelling, and epiglottitis (Figures 1, 2). Imaging also demonstrated diffuse inflammatory changes involving the parapharyngeal soft tissues and significant luminal narrowing at the level of the vocal cords (Figure 2). Extensive phlegmonous changes with obliteration of the soft tissues along the left side of the airway were also noted (Figure 3). Effacement of the left piriform sinus and vallecula was observed, as well as air in the soft tissues surrounding the hyoid bone on the right side (Figure 1).

Otolaryngology was consulted, and the patient underwent transoral incision and drainage of the parapharyngeal abscess under general anesthesia. Approximately 3 mL of purulent fluid was aspirated, and cultures grew Group A Streptococcus (GAS).

The patient’s postoperative course was uneventful, with marked improvement in symptoms over the next 48 hours. Repeat imaging was not deemed necessary as clinical signs of infection resolved. The patient was discharged on a week course of oral amoxicillin-clavulanate. This case highlights the importance of timely diagnosis and management of deep neck space infections.

## Discussion

Although acute pharyngitis is typically viral in etiology, self-limiting, and more common in childhood and adolescence, other causes, including bacterial infections, can occur, with GAS being the most common [6]. In adults, acute pharyngitis due to GAS accounts for only 5%-15% of cases, and the rate steadily decreases after the age of 40 [6]. Since acute bacterial pharyngitis is more prevalent in individuals under 18 years old, the literature predominantly focuses on this age group, with less emphasis on adults. This trend is mirrored by clinicians, who often place GAS pharyngitis at the bottom of their differential diagnosis in adult patients. This case, however, emphasizes the importance of maintaining a high suspicion for GAS pharyngitis, even in adults, given the potential for severe complications such as parapharyngeal abscesses.

In this report, the 43-year-old patient, with no significant past medical history, initially presented with symptoms consistent with acute pharyngitis, which typically resolves on its own within five to seven days, even without antibiotics [7]. However, this patient developed a parapharyngeal abscess, with the infection spreading to multiple head and neck structures, leading to significant airway compromise. This case highlights that, although uncommon, adults can develop severe complications from what initially appears to be a simple viral or bacterial upper respiratory infection. Such complications, including airway obstruction and the need for ICU admission and surgical drainage, underscore the importance of early recognition and intervention.

Another contributing factor to the patient's severe presentation may be co-infection with Group G Streptococcus (GGS), in addition to the culture-confirmed GAS infection. GGS shares many virulence factors with GAS, such as M protein, streptolysin O and S, streptokinase, and pyrogenic exotoxins [8]. Although GGS is part of the normal flora of the human upper airway, it has been recognized as a significant pathogen in deep neck space infections, including parapharyngeal and peritonsillar abscesses. These infections, especially when involving the airway, can lead to life-threatening complications, including upper airway obstruction [8].

Alternative empiric antibiotic regimens for deep neck space infections include piperacillin-tazobactam, ceftriaxone combined with metronidazole or clindamycin with a fluoroquinolone in patients with penicillin allergies [3,5]. Although Group A Streptococcus was isolated in this case, broader sensitivity testing was not available, limiting assessment of potential resistance. Adjunctive therapies can also play a role in managing complications such as airway edema. Corticosteroids have been shown to reduce inflammation and improve airway patency, particularly in the setting of concurrent epiglottitis [1]. Inhalational agents such as racemic epinephrine may provide temporary symptom relief in cases of upper airway obstruction, although their effects may be transient and should be used with caution due to the risk of rebound edema [1].

In our patient, the infection was severe enough to involve the parapharyngeal space, epiglottitis, and significant luminal narrowing at the level of the vocal cords, as seen on imaging. This spread of infection within critical neck structures supports the hypothesis that GGS could have contributed to the patient’s severe clinical course. Furthermore, GGS is more commonly associated with older adults, particularly those with comorbidities such as diabetes mellitus, alcohol abuse or malignancies [9]. While our patient was relatively young and healthy, the pathogenicity of GGS and its role in deep neck infections should be considered even in adults without apparent risk factors. This case highlights the need for high suspicion of severe complications, even in adults without obvious risk factors, and emphasizes the importance of prompt medical and surgical intervention to prevent airway obstruction and other serious outcomes.

## Conclusions

This case underscores the importance of prompt recognition, diagnosis, and multidisciplinary management in addressing deep neck infections, such as parapharyngeal abscesses, even in adult patients without common risk factors. The patient’s favorable outcome highlights the efficacy of a combined approach involving early imaging, appropriate antimicrobial therapy, and surgical drainage in managing such rare but potentially life-threatening conditions. Additionally, this report emphasizes the need for heightened clinical vigilance for bacterial pathogens like GAS and the possible contribution of co-infecting organisms, such as GGS, which may exacerbate disease severity. The insights gained from this case contribute to the broader understanding of deep neck infections and the necessity for timely intervention to prevent complications, including airway obstruction and systemic sepsis.
